# DNA methylome of human neonatal umbilical cord: Enrichment of differentially methylated regions compared to umbilical cord blood DNA at transcription factor genes involved in body patterning and effects of maternal folate deficiency or children’s sex

**DOI:** 10.1371/journal.pone.0214307

**Published:** 2019-05-07

**Authors:** Kenichi Sakurai, Keiko Shioda, Akifumi Eguchi, Masahiro Watanabe, Hidenori Miyaso, Chisato Mori, Toshi Shioda

**Affiliations:** 1 Department of Nutrition and Metabolic Medicine, Center for Preventive Medical Sciences, Chiba University, Chiba, Japan; 2 Center for Cancer Research, Massachusetts General Hospital and Harvard Medical School, Charlestown, MA, United States of America; 3 Department of Sustainable Health Science, Center for Preventive Medical Sciences, Chiba University, Chiba, Japan; 4 Department of Bioenvironmental Medicine, Graduate School of Medicine, Chiba University, Chiba, Japan; University of Bonn, Institute of Experimental Hematology and Transfusion Medicine, GERMANY

## Abstract

The DOHaD (developmental origins of health and disease) hypothesis claims that fetal malnutrition or exposure to environmental pollutants may affect their lifelong health. Epigenetic changes may play significant roles in DOHaD; however, access to human fetuses for research has ethical and technical hurdles. Umbilical cord blood (CB) has been commonly used as an epigenetic surrogate of fetuses, but it does not provide direct evidence of fetal exposure to pollutants. Here, we propose umbilical cord tissue (UC), which accumulates substances delivered to fetuses during gestation, as an alternative surrogate for epigenetic studies on fetuses. To explore the feasibility to examine UC epigenome by deep sequencing, we determined CpG methylation profiles of human postnatal UC by reduced representation bisulfite sequencing. Principal component analysis clearly separated the DNA methylomes of UC and CB pairs isolated from the same newborn (n = 10). Although all UC chromosomes were modestly hypomethylated compared to CB chromosomes, GO analysis revealed strong enrichment of differentially methylated regions (DMRs) at promoter-associated CpG islands in the *HOX* gene clusters and other genes encoding transcription factors involved in determination of the body pattern. DNA methylomes of UC autosomes were largely comparable between males and females. Deficiency of folate during pregnancy has been suggested to affect fetal DNA methylation to cause congenital anomalies. Whereas DNA methylome of UC was not significantly affected by early-gestational (12 weeks) low levels of maternal plasma folate (< 8 ng/ml, n = 10) compared to controls (>19 ng/mL, n = 10), two specific loci of LTR12C endogenous retroviruses in chromosome 12 were significantly hypermethylated in the low-folate group. Our study suggests that UC is useful as an alternative surrogate for studying environmental effects on DNA methylation in human fetuses, compensating CB by providing additional information about epigenetic regulation of genes involved in developmental body patterning and endogenous retroviruses.

## Introduction

Developmental Origins of Health and Disease (DOHaD) is a hypothesis claiming that the early life environment, including fetal to infantile stages of human development, can impact the risk of chronic diseases from childhood to adulthood. Epidemiological studies have been accumulating an increasing amount of evidence supporting the significance of the DOHaD phenomena in human health[1–3]. To explain the delayed-onset effects that become evident after significant periods of latency, recent studies propose that epigenetic mechanisms, especially DNA methylation, may convey memory of stress experiences [4–11]. To examine this hypothesis, quantitative and comprehensive assessment of the DNA methylomes of human fetuses and neonates with associating evidence and/or known history of prenatal stresses may provide important opportunities of evaluating the epigenetic impact of various types of stresses and identifying epigenetic biomarkers for the DOHaD-models of adult diseases.

Despite the importance of DNA methylome profiling of human fetuses and newborn children, access to their tissue specimens is limited due to ethical restrictions and technical risk of affecting normal development. For this reason, non-invasive surrogate tissues of human neonates–namely, placenta, umbilical cord blood (CB), and umbilical cord tissue (UC)–have been playing important roles in estimating epigenetic impact of prenatal stresses. Among these three surrogates, placenta consists of both fetal and maternal cells whereas CB and UC are solely derived from the fetus. Thus, placenta provides unique opportunities to investigate the interface of the fetus-mother interactions for DOHaD research [12, 13]. However, the presence of both fetal and maternal components in placenta may introduce uncertainties in its genomic or epigenomic analyses unless they are carefully separated through painstaking procedures [14]. Although all blood cells in CB are fetal origin, they show significant cell type heterogeneity that strongly impact the outcome of DNA methylome analyses. Taking advantage of the epigenome-wide association study of Boston Birth Cohort, Braid *et al*. have demonstrated the critical importance of correcting DNA methylome data of human CB based on cell type proportion [15]. For example, among infants exposed to antenatal steroids, the number of differentially methylated CpG sites dropped from 127 to only one after controlling for cell type proportion [15], suggesting that it is essential to include blood cell type proportion analysis in CB-based epigenetic studies for adequate correction of the epigenome data for cellular heterogeneity. Although UC also consists of heterogeneous structures–namely, Wharton’s jelly, blood vessels and amnion–the structure of UC is largely identical through the late stages of human gestation and well-maintained among specimens collected from different newborns [16]. Lim *et al*. reported that DNA methylation at the promoter of *PEG10* paternally expressed imprinted gene, which plays critical roles in placenta formation [17], was augmented to suppress mRNA expression in UC of newborns belonging to a group of low birth weight [18]. Godfrey *et al*. have shown that augmented DNA methylation in UC at the retinoid X receptor-α (*RXRA*) gene promoter was associated with an increase in sex-adjusted childhood fat mass at 6 or 9 years of age as well as a greater amount of maternal carbohydrate intake during early pregnancy [19]. These studies support the potential usefulness of UC, without separation of its heterogeneous structures, for epigenetic studies in the context of DOHaD.

To obtain further insights into the use of UC as epigenetic surrogate of neonates, in the present study we compared DNA methylomes of UC and CB obtained from the same neonate. We also examined UC DNA methylomes between groups of high and low maternal plasma folates as well as effects of neonatal sex. Results of the current study suggest that UC may provide a unique opportunity to examine epigenetic effects on the body type-determinant *HOX* gene clusters that might be masked in CB due to terminal differentiation to the blood cell lineage. UC may also be useful for studying environmental impact on epigenetic suppression of the endogenous retroviruses.

## Materials and methods

### Human study subjects

Pregnant women were selected from the Chiba study of Mother and Child Health (C-MACH), a birth cohort study designed for multi-omics analyses of the genetic and environmental impact on children’s health [20]. The participants were recruited from February 2014 to June 2015. Consent to participate in the C-MACH was obtained from 433 women, and 376 of them completed questionnaire in the early gestational period. The questionnaire-completed women were subjected to the following exclusion criteria: experience of abortion or pre- or post-term delivery; maternal smoking; or treatment for infertility. From the three local cohorts of the C-MACH (Onodera, Yamaguchi, and Aiwa cohorts), all subjects of the present study were selected from the Onodera cohort. For comparison of the UC and CB DNA methylomes, 10 subjects were selected for availability of both frozen specimens of UC and CB (5 male neonates and 5 female neonates; S1 Table). To study association between maternal folate deficiency and UC DNA methylome, two groups of 10 participants whose plasma folate level at 12-weeks gestation was lower than 8 ng/ml (low folate group) or greater than 19 ng/mL (high folate group) were selected with matched age, pre-pregnancy body mass index, and gestational weight gain (S1 and S2 Tables).

### Ethics

The study protocol was approved by the Biomedical Research Ethics Committee of the Graduate School of Medicine, Chiba University (ID 451: application date 8 November 2013; ID 462: application date 4 December 2013; ID 502: application date 28 May 2014). Informed consent forms for participating for C-MACH were obtained from the study participants who were pregnant females at gestational age at <13 weeks. The mothers consented to give umbilical cords and cord blood at delivery for studies.

### Genomic DNA extraction

Intact whole umbilical cords were chilled on ice immediately after delivery and transferred from the delivery room of the participating hospitals to the C-MACH laboratory at the Chiba University under cooling within 12 hours. Cords were then washed with ice-cold PBS to remove maternal blood or CB, and blood-free UC were cut into rings and snap frozen in liquid nitrogen. The UC slices were stored at –80°C until analysis. The UC slices were ground to fine power using the Coolmil tissue grinder (Tokken, Chiba, Japan) chilled by liquid nitrogen. Genomic DNA was extracted from the frozen powder using the NucleoSpin Tissue kit (Takara Bio, Shiga, Japan) and quantified using NanoDrop (NanoDrop Technologies, Wilmington, DE, USA) and QuantiFluor dsDNA System (Promega, Madison, WI, USA).

CB was collected from intact whole umbilical cords, and mononuclear cells were collected using the BD VacutainerR CPTTM tubes (Becton, Dickinson and Company, Franklin Lakes, NJ, USA) according to the manufacture’s instruction. Genomic DNA was extracted and quantified as described above.

### Reduced representation bisulfite sequencing (RRBS)

RRBS is a bisulfite-based deep sequencing technique for genome-wide interrogation of CpG dinucleotide methylation focusing on promoter-associated CpG islands [21]. Genomic DNA of UC or CB (100 ng) was digested by MspI restriction enzyme and subjected to RRBS library construction using Ovation RRBS Methyl-Seq System 1–16 (Nugen, San Carlos, CA, USA) following the manufacturer’s instruction. Bisulfite conversion of the Ovation gDNA library was performed using the Epitect Fast Bisulfite Conversion kit (Qiagen, Germantown, MD, USA). Greater than 99% bisulfite conversion efficiency was confirmed using unmethylated lambda DNA spike-in as described in our previous study [22]. Barcoded RRBS libraries were quantified using the KAPA qPCR quantitation kit for Illumina platform (Roche, Pleasanton, CA, USA) for equimolar pooling and subjected to multiplex deep sequencing (75 nt, single read) using the Illumina NextSeq deep sequencer (Illumina, San Diego, CA, USA) and the Custom Primer 1 provided by the manufacturer of the library construction kit (NuGen). To avoid batch effect, we performed the entire process of genomic DNA isolation, deep sequencing library preparation, and sequencer run without creating any batches. Thus, all the 20 genomic DNA samples involved in the CB-UC comparisons were processed together. Similarly, all the 20 UC genomic DNA samples involved in the analyses of sex and folate effects were processed as a single batch. Barcoded deep sequencing libraries for the 20 CB-UC comparisons were pooled, and deep sequencer runs were repeated with the same pool until necessary numbers of reads were obtained. The separate, 20 UC libraries were also barcoded, pooled, subjected to repeated sequencer runs. The numbers of uniquely mapped reads (S1A Fig) do not show any significant tendencies linking the minor variations in them to biologically relevant attributes of the samples or analytical outcomes (*See* S1 Table for attributes of samples, including sex and maternal blood folate level).

### Bioinformatics and statistics

The FASTQ raw read sequence data were subjected to adaptor trimming and low-quality reads exclusion using the trimGalore! (version 0.4.4_dev) RRBS quality control tool (Bioinformatics Group, the Babraham Institute, Cambridge, UK) with the following command line: “trim_galore—quality 20 -a AGATCGGAAGAGC—fastqc raw_*fastq_file*.*fastq*” executed in a MacPro computer (MacOS 10.12.6). The resulting trimmed FASTQ files were processed further for removal of the diversity adaptors using the trimRRBSdiversityAdaptCustomers.py python script (version 1.11) provided by the manufacturer of the RRBS library construction kit (NuGen). The trimmed FASTQ files generated by the four NextSeq sequncer lanes were then merged and subjected to RRBS read alignment to human GRCh38/hg38 reference genome sequence using Bismark (version 0.18.1_dev) [23] with the Bowtie2 (version 2.3.4) [24] backend aligner with the following command line: “bismark—bowtie2 -p 4—multicore 4 *directory_to_Bismark_genome_folder trimmed_fastq_file*.*fastq*.” The resulting Bismark BAM files were subjected to removal of reads with signs of incomplete bisulfite conversion using the filter_non_conversion script, which was included in the Bismark software package. Information on DNA methylation at each CpG site was extracted using the bismark_methylation_extractor script included in the Bismark package using the following command line: “bismark_methylation_extractor—single-end—multicore 14—bedgraph—buffer_size 40G *bam_filename*.*bam*.” Data visualization and statistical analyses of the RRBS data, including identification, localization, and annotation of differentially methylated regions (DMRs), were performed using the BiSeq (version 1.18.0) R/bioconductor package [25]. Gene ontology analysis of the DMR-associated protein-coding genes was performed using the DAVID server [26]. To inspect DNA methylation profiles along locations of genes, BEDGRAPH files generated by Bismark were visualized using the integrative genomics viewer [27]. Standard statistical tests (Student’s *t-*test, Fisher’s exact test, and Wilcoxon rank-sum test, and power analysis) were performed using R (package version 3.4.2). Numbers of the uniquely mapped reads generated from the libraries for comparison of UC and CB were 4.5–9.5 million, and from the UC libraries for analyses of sex or folate effects were 15–26 million (S1A Fig).

### Bisulfite pyrosequencing

Bisulfite conversion of genomic DNA and quality control of the conversion efficiency was performed as described in our previous study [22]. DNA methylation of the multiple copies of the LTR12C endogenous retrovirus was determined by EpigenDx (Hopkinton, MA, USA) using custom pyrosequencing assays ADS5716 (interrogated sequence = GTYGG/AAGTYGGTTTTTTTAGTTTGTAGGGAGG, containing one known SNP and two CpG sites [shown as YG]) and ADS5717 (interrogated sequence = TTTTYGTYGATTGTT/GTTAGTTGTTTTTTYGYGGGGTAGGGTTYGGGATTTGTAGTTYGTTATGTTTGAG, containing one known SNP and six CpG sites).

## Results

### Global differences in the DNA methylomes of UC and CB

As an initial step of exploring the feasibility of using UC as an alternative surrogate of human newborns for epigenetic studies, we determined DNA methylomes of CB and UC obtained from the same newborn by RRBS (n = 10; 5 males and 5 females) and compared their global quality control profiles. For each CB or UC library, approximately 5 million RRBS reads were uniquely mapped to the human GRCh38/hg38 reference genome sequence (S1A Fig). When combined data sets of CB or UC libraries were analyzed, each CpG site was interrogated with at least 10 informative reads, and the CpG coverage profiles were comparable between the CB and UC combined data sets (coverage peak: Log_10_1.3 ≈ 20; S1B Fig). Methylation of each CpG site in the combined CB and UC data sets showed comparable profiles with slightly reduced frequencies of strongly methylated CpG sites in UC compared to CB (S1C Fig). These results indicate that RRBS analysis of CpG methylation can be performed for the genomic DNA isolated from human UC specimens with quality control parameters comparable to those of human CB specimens.

**Fig 1:**
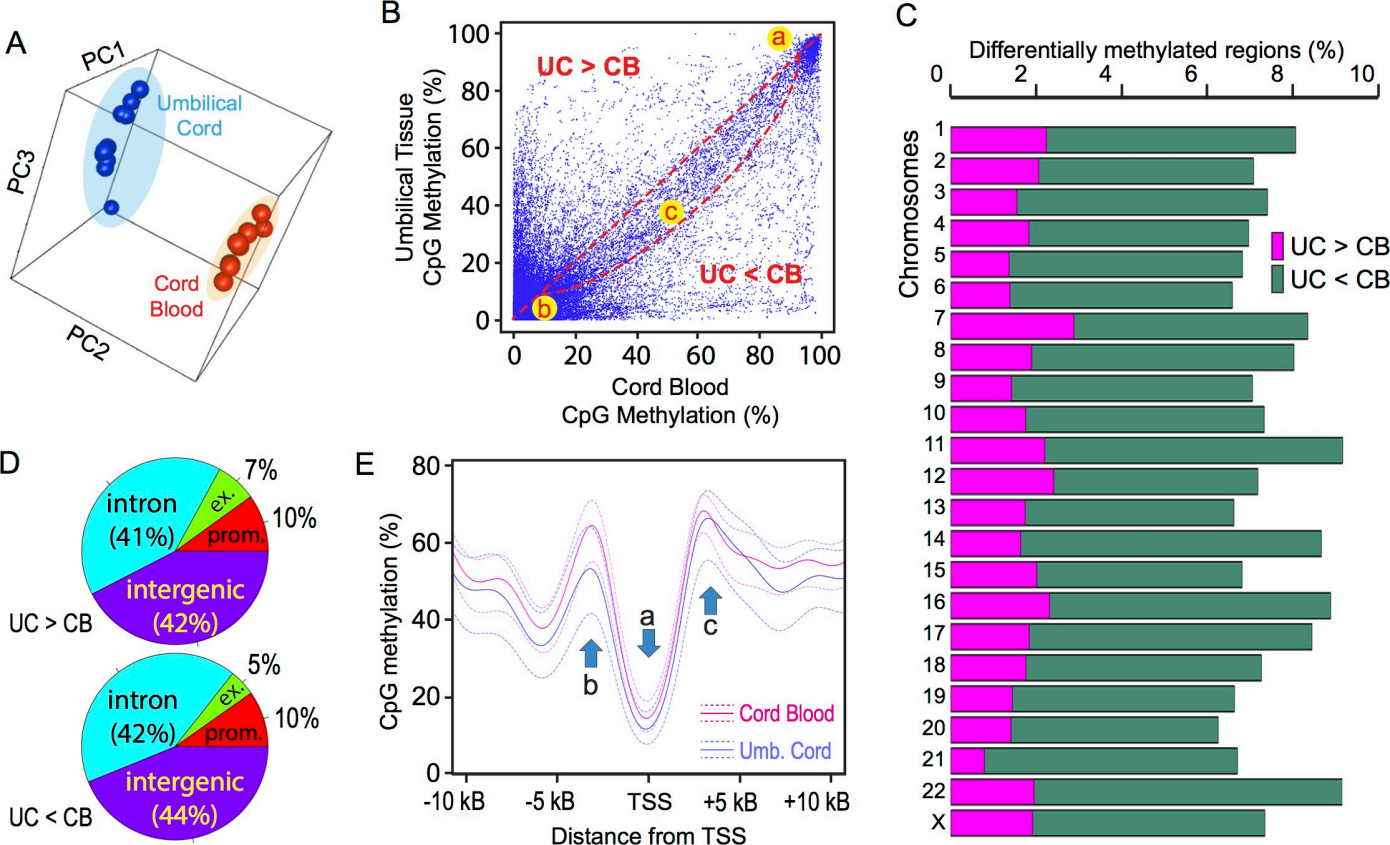
Comparison of DNA methylomes between human umbilical cord tissue (UC) and umbilical cord blood (CB). DNA methylomes of UC and CB obtained from the same newborn (n = 10, 5 males and 5 females) were determined by the reduced representation bisulfite sequencing (RRBS). (A) Principal component analysis of 10 DNA methylomes of UC (*blue dots*) and CB (*red dots*). (B) X-Y plot profiling of combined CB data (*x-axis*) and UC data (*y-axis*) for methylation at all RRBS-interrogated autosomal CpG sites. High density areas *a* and *b* represent CpG sites with strong hyper- and hypo-methylation, respectively, in both UC and CB. The diagonal, crescent-shape high density area *c* includes CpG sites with reduced methylation in UC compared to CB. (C) Relative numbers of differentially methylated regions (DMRs) in all RRBS-interrogated CpG sites calculated for each chromosome. *Magenta*, hypermethylated in UC than CB; *green*, hypomethylated in UC than CB. (D) Locations of DMRs in the functionally distinct regions in human genome. *Prom*., promoter; *ex*., exon. (E) DNA methylation profiles of UC (*blue*) and CB (*red*) near the transcription start sites (TSS). The solid lines indicate mean, and the dotted lines indicate 25% or 75% quantiles. Arrow *a* indicates hypomethylation at the TSS; *b* and *c* indicate pre-TSS and post-TSS hypermethylation, respectively.

Principal component analysis (PCA) clearly separated DNA methylomes of UC and CB specimens (Fig 1A; p < 0.00001 along PC2, two-sided *t*-test). Because PCA did not reveal significant effects of the children’s sex in each tissue type, the subsequent analyses were performed with combined data sets of UC or CB libraries including both male and female newborns. X-Y plotting of CpG methylation in UC and CB genomes revealed a high-density region of dots reflecting very strong methylation in both UC and CB (Fig 1B, region *a*). Another high-density region (*b*) indicated a large number of strongly demethylated CpG sites in both UC and CB. The presence of these strongly methylated or demethylated CpG sites shared by UC and CB agreed with the dichotomy pattern of CpG methylation shown in S1C Fig. The third high-density region (*c*) aligned along the diagonal line connecting the regions *a* and *b* showed a discernible bias towards the bottom, resulting in the crescent shape of this region. This bias indicated that CpG sites with modest strengths of methylation were generally more hypomethylated in UC than CB in the entire genome. When the individual chromosomes were examined, all chromosomes had greater sizes of regions hypomethylated in UC compared to CB than regions hypermethylated in UC (Fig 1C), indicating that the observed hypomethylation in UC was a general tendency observed throughout the genome. For both DMRs hyper- or hypo-methylated in UC compared to CB, most DMRs were located in either intergenic regions or introns while 10% of them were associated with promoters of protein-coding genes (Fig 1D). Only 7 or 5% of all DMRs hyper- or hypo-methylated in UC were located within the exons, respectively. Detailed examination of the promoter-associated DMRs revealed that the hypomethylation at the transcription start sites (TSS) and the hypermethylation at immediately downstream of the TSS were comparable between UC and CB. In contrast, DNA methylation at immediately upstream of the TSS was weaker in UC than CB (Fig 1E). Thus, although the overall numbers of DMRs at promoters were comparable between those hyper- and hypo-methylated in UC, promoter hypomethylation in UC tended to impact the region immediately upstream of the TSS more strongly than other regions. This location-specific DNA hypomethylation in UC than CB around the TSS implied involvement of specific mechanisms of epigenetic regulation.

### Functional implications of the DMRs between UC and CB

**Fig 2:**
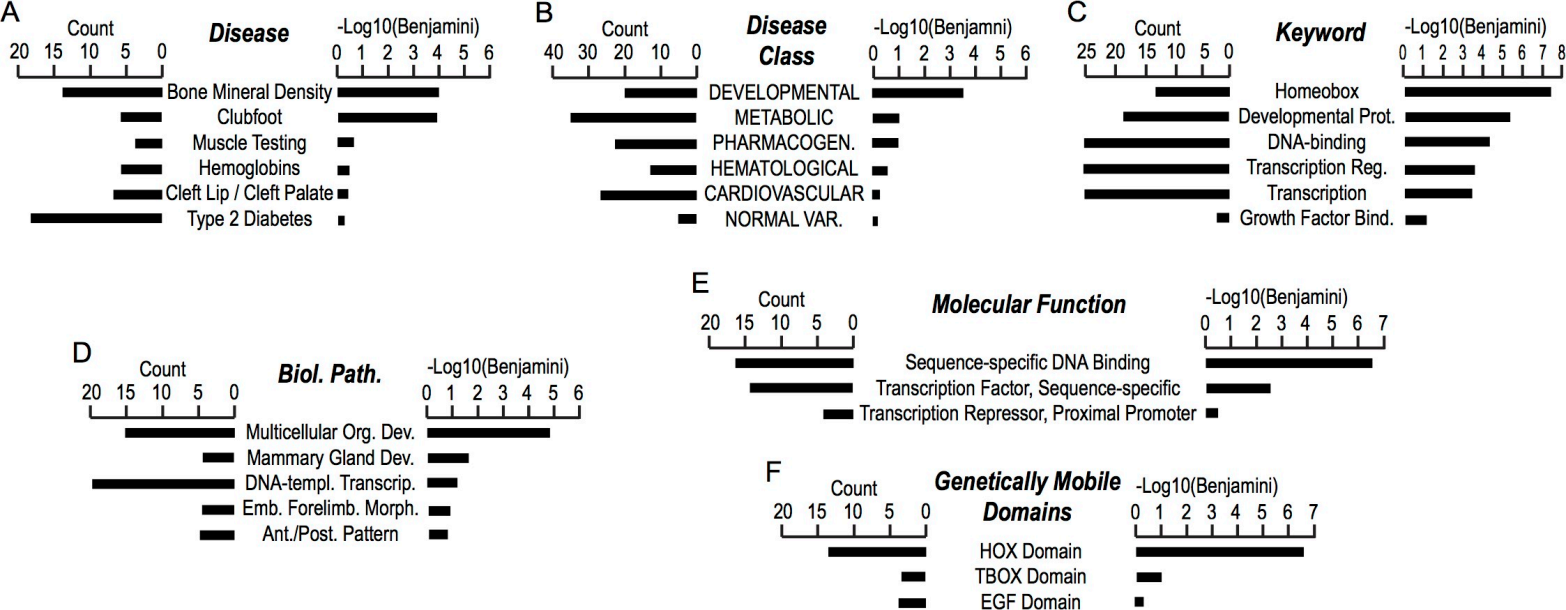
Gene ontology analysis of DMRs hypermethylated in UC than CB. Each panel shows GO terms, counts of DMRs, and strength of statistical significance (-Log10 of Benjamini-corrected *p*-values).

**Fig 3:**
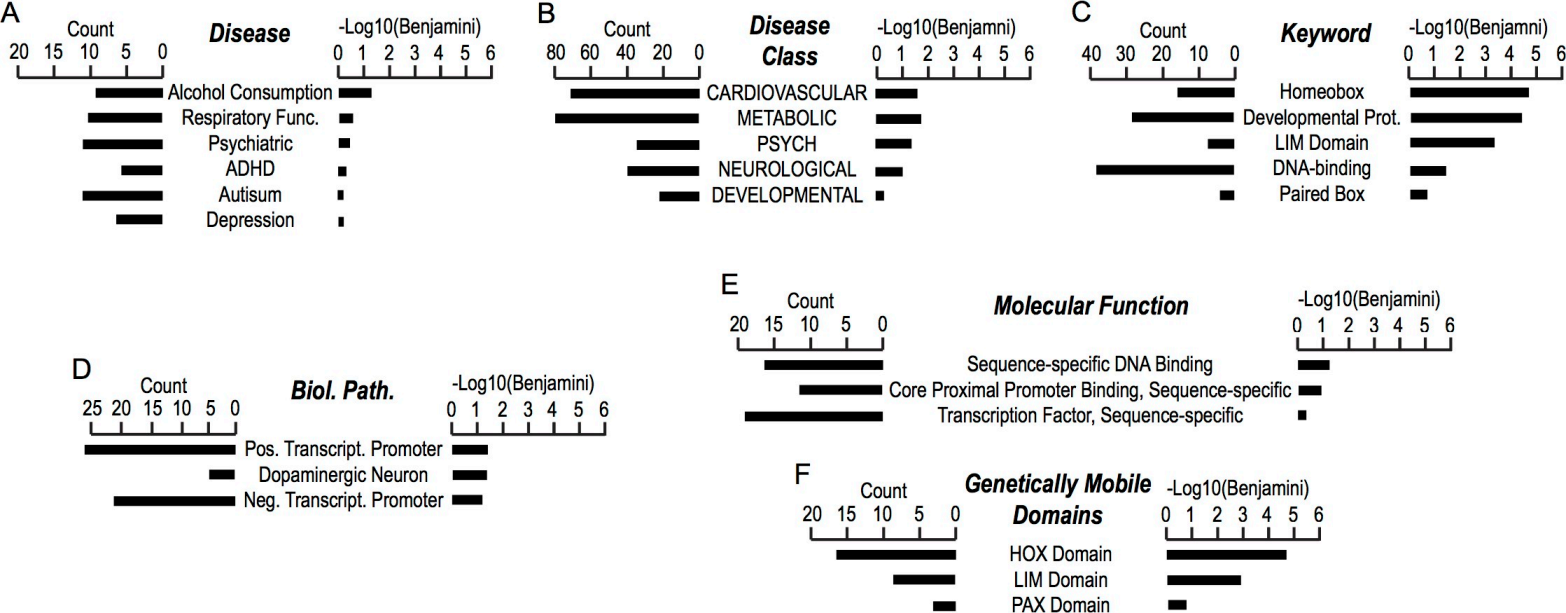
Gene ontology analysis of DMRs hypomethylated in UC than CB. Each panel shows GO terms, counts of DMRs, and strength of statistical significance (-Log10 of Benjamini-corrected *p*-values).

Our RRBS analysis revealed that DMRs hypermethylated in UC than CB were associated with promoters of 102 protein-coding genes (S3 Table). Gene ontology analysis of these genes identified significant associations with diseases affecting bones (*e*.*g*, aberrant bone mineral density or clubfoot; Fig 2A) or development (Fig 2B and 2D). A strong association was observed with DNA-binding transcription factors, especially with the homeobox domain-containing HOX genes (Fig 2C and 2E), an evolutionarily conserved family of genes encoding DNA-binding transcription factors whose roles are essential for developmental body patterning and cellular differentiation [28]. Similarly, we identified DMRs hypomethylated in UC than CB associating promoters of 281 protein-coding genes (S3 Table). Although these UC-hypomethylated DMRs showed only modest associations with diseases (Fig 3A, 3B and 3D), they again showed a very strong association with the HOX genes (Fig 3C, 3E and 3F). Taken together, our gene ontology study suggested that DMRs between UC and CB are strongly associated with genes encoding DNA-binding transcription factors that play important roles during the prenatal development of human.

**Fig 4:**
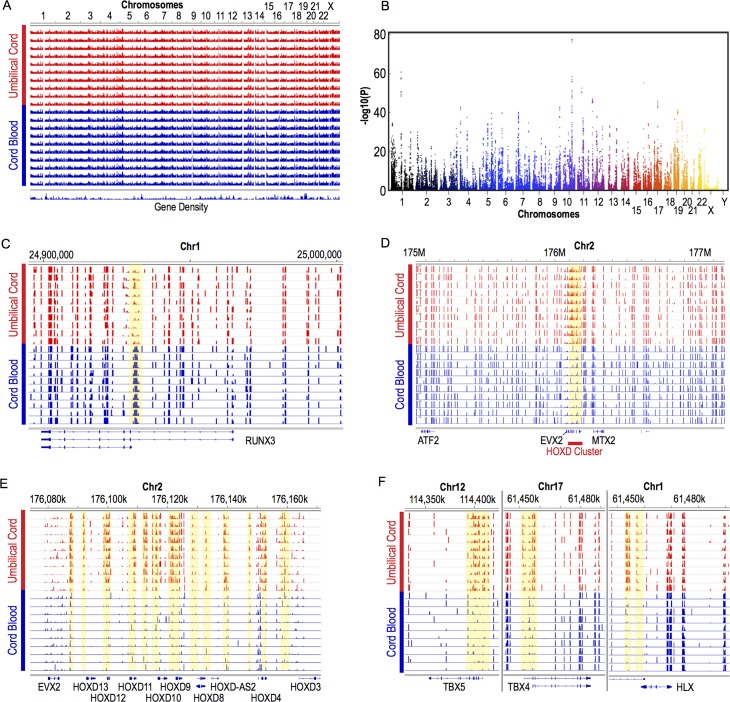
Visualized DMR profiles of UC and CB. DNA methylation profiles of 10 UC (*red*) and 10 CB (*blue*) for the whole genome (A, B), the *HOXD* gene cluster (D, E) or individual genes (C, F) were visualized using the integrative genomics viewer (A, C-F) or as a Manhattan plot of DMRs between UC and CB (B). Each bar in panels A and C-F indicates degree of DNA methylation; y-axis scale of all tracks is 0–100%, and yellow shades indicated DMRs (C, hypomethylated in UC than CB; D-F, hypermethylated in UC than CB).

To examine the specificity of DMRs associating DNA-binding transcription factors, we performed visual inspection of DNA methylomes of the individual UC or CB libraries (Fig 4). Inspection of the RRBS deep sequencing tracks for global DNA methylomes of all UC and CB specimens by the integrative genome viewer revealed nearly identical profiles with no discernible large DMRs at the chromosomal level (Fig 4A), supporting the comparable overall quality of the RRBS data of each sample. Nonetheless, Manhattan plot analysis, which efficiently detects statistically significant differences between groups of whole genomes at short sequences even in the single nucleotide level, demonstrated existence of multiple DMRs between UC and CB (Fig 4B). Multiple DMRs were detected in all chromosomes although their densities varied among different chromosomes and regions.

The DMR associating *RUNX3*, which encodes a transcription factor involved in growth and differentiation of gastric epithelial cells [29], was strongly methylated in CB (Fig 4C, yellow band). The long and short RUNX3 mRNA transcripts are transcribed from the P1 and P2 promoters, respectively, in a tissue-specific manner [30]. A strong association between hypermethylation of the P2 promoter, which occurs at the identical location of the DMR shown in Fig 4C, and the risk of gastric cancer has been confirmed by a meta-analysis of 16 studies [31]. Outside this P2 promoter DMR, DNA methylation profiles within and near the *RUNX3* gene were largely comparable across all UC and CB specimens. These results indicate that the UC-CB DMR associating *RUNX3* is specific, presumably involved in differential expression of this gene in UC and CB. The *RUNX3*-associating DMR thus serves as a representative example of promoter-associated DMRs hypomethylated in UC.

Human HOXD cluster consists of nine *HOXD* genes and lies between two topologically associating domains (TADs) that control stage- and location-specific expression of *HOXD* gene subsets during body patterning of development [32]. The HOXD cluster was specifically hypermethylated in UC specimens (Fig 4D). More detailed inspection of the HOXD cluster (*HOXD13*—*HOXD3*) revealed multiple independent small DMRs hypermethylated in UC whereas not a single instance of DMR hypomethylated in UC was observed (Fig 4E). Thus, the DMR of the HOXD cluster in UC involves coordinated hypermethylation at multiple locations.

The T-box transcription factors TBX4 and TBX5 expressed in muscular connective tissue play critical roles in patterning of muscles and tendons [33]. In humans, mutations of *TBX4* causes Small Patella syndrome, which involves skeletal dysplasia [34]. *TBX5* mutations lead to Holt-Oram syndrome, which is characterized by forelimb musculo-skeletal defects [35, 36]. Promoters of both *TBX4* and *TBX5* genes located on distinct chromosomes were specifically hypermethylated in UC compared to CB (Fig 4F). Similarly, specific hypermethylation in UC was observed at the promoter of HLX, which encodes a homeobox transcription factor involved in development of hematopoietic, hepatic, and digestive tract lineages [37] (Fig 4F). Taken together, visual inspections of the RRBS deep sequencing tracks confirmed specific and significant differential methylation of genes encoding transcription factors involved in developmental body patterning or lineage-specific cellular differentiation.

### Effects of neonatal sex on DNA methylome of UC

To examine effects of gestational environment on DNA methylome in UC, we determined DNA methylomes of UC obtained from a new group of 20 newborns (7 males and 13 females) by RRBS with 15–25 million uniquely mapped reads (S1A Fig). UC specimens in this group had no overlap with the UC examined for comparisons with CB, and they were associated with data describing neonates (route of delivery, gestational age, sex, height, weight) and mothers (plasma folate and vitamin B12 levels at 12-weeks of gestation, age, height, weight, body mass index, and parity) (S1 Table). Although all mothers were non-smokers, information on the paternal smoking was also obtained.

**Fig 5:**
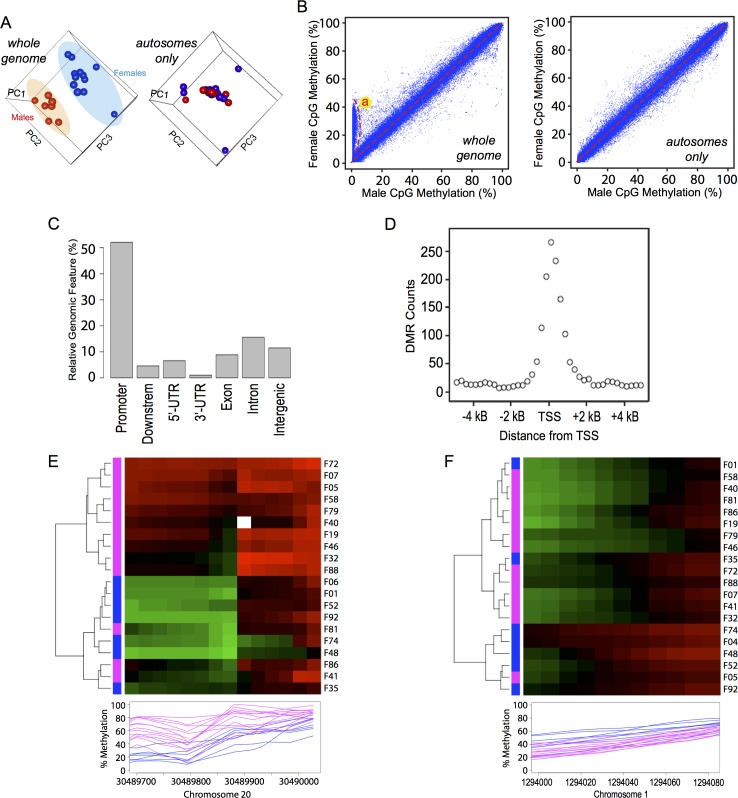
Comparison of DNA methylomes of UC between males and females. DNA methylomes of UC (7 males and 13 females) were determined by RRBS. (A) Principal component analysis of whole genome (*left*) or autosomal (*right*) UC DNA methylomes of males (*red dots*) and females (*blue dots*). (B) X-Y plot profiling of combined male (*x-axis*) and female (*y-axis*) whole genome (*left*) or autosomal (*right*) DNA methylomes. The crescent-shape area *a* indicates female-specific hypermethylation regions shown in the whole-genome analysis but not in the autosomal analysis. (C, D) Locations of autosomal DMRs in the functionally distinct regions in human genome (C) or near the TSS (D). (E, F) CpG sites methylation at two representative DMRs in the individual UC samples; hypomethylated in males (E) or hypermethylated in males (F). The heatmap (*top*) and the line plot (*bottom*) show CpG methylation (0–100% scale) for the chromosomal coordinates indicated at the bottom. Colors of the vertical bar and the line plots indicates sex (*blue*, *males*; *magenta*, *females*). An CpG whose methylation state was not determined due to insufficient data quality is indicated with a white blank.

To establish a foundation of the use of UC as epigenetic surrogates of neonates, we first determined effects of neonatal sex on DNA methylome. Whole-genome PCA analysis involving both autosomal and sex chromosomes clearly separated DNA methylomes of males and females (Fig 5A, left; *p* < 0.00001 along PC3, two-sided *t*-test). X-Y plotting of CpG methylation in combined whole-genome DNA methylomes revealed a female-specific high-density region of dots reflecting the strongly methylated, an inactive copy of X chromosome (Fig 5B, left, region *a*). Male-specific high-density region of dots reflecting the presence of Y chromosome was not obvious, presumably due to the very small number of CpG sites in human Y chromosome [38]. When sex chromosomes were excluded from the analysis, both PCA (Fig 5A, right) and X-Y plotting (Fig 5B, right) indicate the absence of significant sex-dependent global differences in DNA methylome (Fig 5A; p > 0.9 along PC axes, two-sided *t*-test).

We next examined statistically significant, autosomal DMRs between males and females. We identified 126 DMRs; 45 and 81 of them were hyper- or hypo-methylated in females compared to males, respectively (S3 Table). The majority of the DMRs (~52%), either hyper- or hypo-methylated in males compared to females, were associated with promoters of known protein-coding genes (Fig 5C). Distribution of these promoter-associated DMRs showed a sharp peak within 1 kbp before and after the TSS (Fig 5D). Despite the strong enrichment of the DMRs to promoters or functional elements of genes (promoters + 5’-UTR + 3’-UTR + Exons ≅ 70% of all DMRs), gene ontology analysis did not identify biological terms with statistically significant enrichment, presumably due to the small number of DMRs. Heatmaps of representative DMRs hyper-methylated in females (Fig 5E) or hypo-methylated in females (Fig 5F) confirm the sex-dependent differential methylation in the individual UC specimens. Taken together, our RRBS data show that autosomal DNA methylome of UC is largely comparable between males and females in the global scale. A relatively small number of the sex-dependent DMRs are strongly enriched around TSS of protein-coding genes, which might be involved in the sex differences in UC.

We confirmed the effects of sex chromosomes on the DNA methylome analysis with the UC and CB data sets examined for differences between these two types of cells (Figs 1–4). 3-D PCA plots of whole-genome DNA methylomes including sex information (S2A Fig) show weak but statistically significant separation between males and females among both UC and CB. Thus, in the UC oval, *dark blue* dots are males, and *green dots* are females; *p* = 0.016 along PC4, two-sided *t*-test. In the CB oval, *red* dots are males, and *orange* dots are females; *p* < 0.001 along PC3, two-sided *t*-test. Separation between UC and CB is obvious (*p* < 0.00001 along PC2, two-sided *t*-test). In contrast, autosomes-only analysis did not separate males and females among either UC or CB (two-sided *t*-test *p* = 0.115 and 0.069 along PC3 for UC and CB, respectively). The strong separation between UC and CB was largely unaffected (*p* < 0.00001 along PC2, two-sided *t*-test). We generated X-Y plots of DNA methylomes of males (*x-axis*) and females (*y-axis*) for all UC and CB combined (S2B Fig), UC only (S2C Fig), and CB only (S2D FiG) for the whole genome (*red dots*), autosomes only (*blue dots*), and whole genome-autosomes merged (mostly *purple dots*). Reproducing the female-specific DNA hypermethylation observed with the UC-only data set (Fig 5B), whole-genome analysis of both UC and CB involved in the UC-CB comparison detected female-specific DNA hypermethylation whereas autosome-only analysis did not (S2C and S2D Fig, *green ovals*). Merged images of the whole-genome and autosome-only analyses clearly visualize the female-specific DNA hypermethylation observed with the whole-genome analysis but not the autosome-only analysis as *red d*ots in the *green ovals*. Due to the significant differences between UC and CB (*See* Fig 1), X-Y plots combining both types of samples (S2B Fig) were unable to detect the DNA hypermethylation in females specific to the whole-genome analysis.

### Association of maternal plasma folate at 12-weeks of gestation with DNA methylome of UC

**Fig 6:**
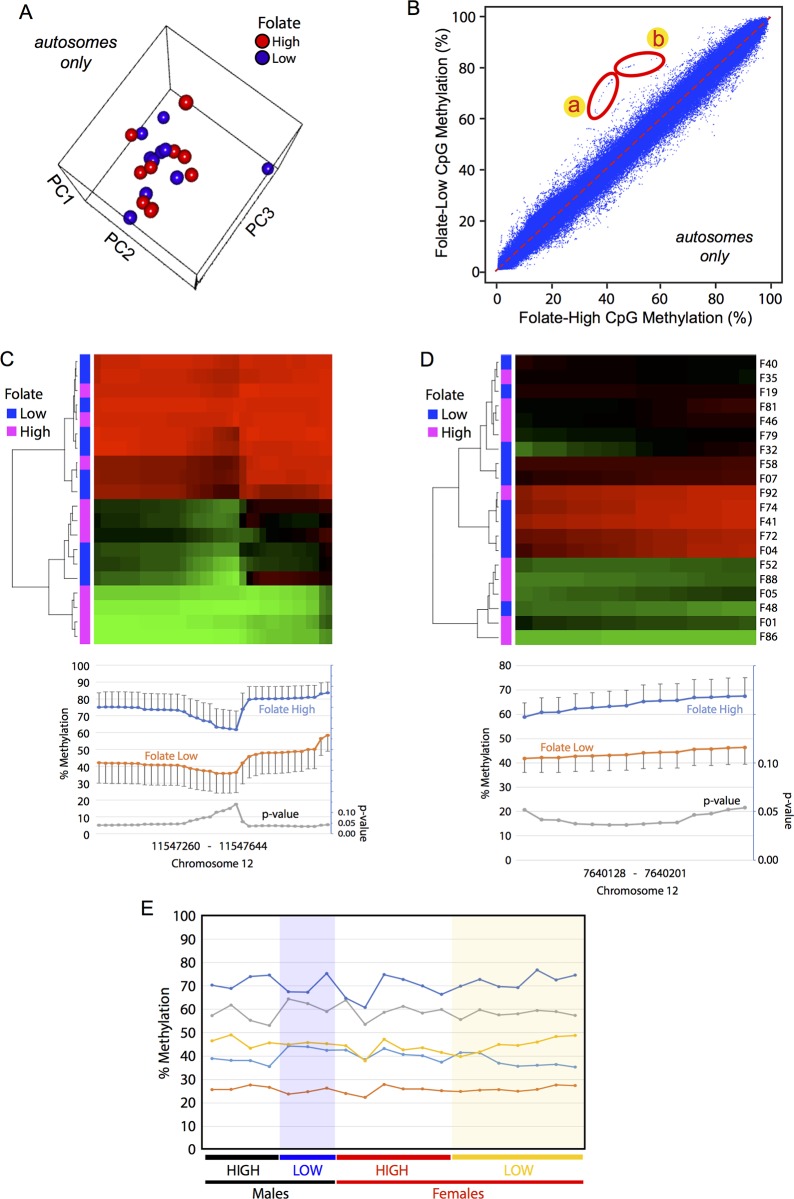
Comparison of autosomal DNA methylomes of UC between maternal high- and low-level plasma folate at 12-weeks gestation. Autosomal DNA methylomes of UC (n = 10 for each of the high- and low-folate groups) were determined by RRBS. (A) Principal component analysis of the individual methylomes. (B) X-Y plot profiling of combined high-folate (*x-axis*) and low-folate (*y-axis*) groups. The circled areas *a* and *b* indicate DMRs, whose detailed CpG methylation profiles are shown in panels (C) and (D), respectively. (C, D) CpG sites methylation at two DMRs circled in panel (B). These DMRs are hypermethylated in folate-low group and corresponded to two copies of the LTR12C endogenous retrovirus located at distinct regions in chromosome 12. The heatmap (*top*) and the line plot (*bottom*; mean ± SEM, n = 10) show CpG methylation for the chromosomal coordinates indicated at the bottom. The *p-*values of two-sided *t-*test calculated for each CpG site are also shown in the line plot. (E) DNA methylation of the LTR12C endogenous retrovirus determined by bisulfite pyrosequencing targeting five CpG sites shown in different colors. Primers for the PCR amplification and pyrosequencing read annealed to the common sequence of the multiple copies of LTR12C existing in the human genome.

To explore the feasibility of the use of UC DNA methylome for evaluation of epigenetic effects of maternal nutrition during gestation, we attempted to detect UC DMRs between children whose mothers’ plasma folate levels at 12-weeks gestation were less than 8 ng/ml (low folate group) or greater than 19 ng/mL (high folate group) (S2 Table). These two groups did not show significant differences in characteristics of the children (route of delivery, gestational age, sex, height, weight) or the mothers (plasma vitamin B12 level at 12-weeks of gestation, age, height, weight, body mass index, and parity) (S2 Table). Autosomal PCA analysis did not separate DNA methylomes of the low- and high-folate groups (Fig 6A), and X-Y plotting of autosomal CpG methylation showed largely identical DNA methylation profiles as well (Fig 6B). However, detailed examinations of the X-Y plot identified CpG sites that are methylated in the low-folate group far more strongly than the high-folate group (Fig 6B, regions *a* and *b*). Annotation analysis revealed that these CpG sites belonged to two independent, solitary copies the LTR12C endogenous retrovirus both in chromosome 12: 11547110–11548392 in the chromosomal band 12p13.2 (Fig 6B, region *a*) and 7639685–7641018 in 12p13.31 (Fig 6B, region *b*). Heatmap analysis of all CpG sites and UC specimens confirmed the statistically significant differential DNA methylation between the low- and high-folate groups (Fig 6C and 6D correspond to regions *a* and *b* of Fig 6B, respectively). Because the Dfam database of the repetitive elements identifies 1,873 copies of LTR12C in human genome [39], we amplified the common sequence of LTR12C from bisulfite-converted UC DNA and determined methylation of five CpG sites therein by pyrosequencing. This bisulfite-pyrosequencing analysis of the overall LTR12C did not detect differences between the low- and high-folate groups in males or females (Fig 6E). These results suggest that the observed association between the maternal plasma folate level during early pregnancy and DNA methylation of the LTR12C endogenous retrovirus is specific to the two copies of the virus in chromosome 12.

## Discussion

Maternal malnutrition or exposure to environmental toxicants during pregnancy may affect epigenetic regulation of genes involved in fetal development [40, 41]. Even when the affected children do not show apparent disorders at the time of birth or during their early stages of life, cryptic epimutations may significantly increase risk of the DOHaD-type adult diseases [42]. Epigenetic analyses of newborns in the context of systematic toxicological assessments of human pregnancy such as C-MACH [20] may provide important information about how the maternal environment can affect the lifelong health of their children through epigenetic reprogramming [43, 44]. However, opportunities of sampling tissues directly from human neonates for epigenetic research purposes are limited due to ethical and technical hurdles. Therefore, adequate surrogates of human neonatal tissues for epigenetic studies are desired.

CB is the blood collected from the umbilical cord after birth, and it has been commonly used as an epigenetic surrogate of neonates [45]. CB is composed of hematopoietic stem cells as well as terminally differentiated erythrocytes or mono-nucleated blood cells originated exclusively from the child without contributions of maternal cells [46]. Different types of cells found in CB have distinct epigenetic profiles [47], and the heterogeneous cellular composition of CB seems highly sensitive to various stresses [15]. Therefore, epigenetic changes observed with CB may reflect altered cellular composition rather than epigenetic reprogramming or epimutations in each type of cells. For this reason, CB-based epigenetic analyses must be corrected based on cell type population or otherwise may suffer from unacceptable high levels of type I errors, as demonstrated recently by Braid *et al*. [15]. Despite the common use of CB as an epigenetic surrogate of neonates, several published studies involve the correction of epigenetic data based on cell type population of CB specimens using microarray [48–50], and the requirement of such correction in the future studies may introduce significant complexity into CB-based epigenetic studies using other methods. Recently, Lin *et al*. reported the cell-type specific DNA methylation profiles of UC tissue, providing an open source panel for estimation and adjustment of cellular heterogeneity in human UC and CB [51]. Their work, together with our current study, can broaden the arsenal of surrogate tissue utilization in future genome-wide epigenetic studies on human neonates. Another disadvantage of CB in the context of toxicological investigations is that lipophilic toxic substances, to which mothers were exposed during pregnancy, are not typically retained for long periods of time, either in its cellular or plasma components. In contrast, UC effectively accumulates lipophilic toxic substances, so the history of maternal exposure to toxicants during pregnancy can be objectively assessed [52]. For example, our previous study detected various species of polycarbonated biphenyls (PCBs) in UC but not in CB whereas all toxicants detected in CB were also detected in UC [53]. Therefore, CB-based toxico-epigenomic analyses are dependent on other sources of information about the history of exposure such as questionnaire, which may not necessarily be accurate or quantitative.

The majority of UC consists of mesenchymal cells (Wharton’s jelly) as well as cells of two arteries and one vein. The structure of sliced rings of UC seems to be consistent throughout the length of UC as well as the course of pregnancy[16]. Our previous study demonstrated greater retention of PCBs in UC than CB [53]. Although the present study is the first systematic report of genome-wide DNA methylome analysis of UC, a few previous studies showed changes in UC DNA methylation at specific genes [18, 19]. Thus, UC seems to have various advantages as an epigenetic surrogate of human neonates although further studies are necessary to establish its merits and drawbacks compared to other surrogate tissues such as CB, placenta, or amniotic fluid.

Because of the rubber-like elasticity and a very high content of high-molecular-weight carbohydrate polymers of UC, extraction of its DNA and CpG methylation analysis have been difficult. Because the carbohydrate polymers co-precipitate with DNA in a non-specific manner with or without CpG methylation, precipitation-based methods of DNA methylation analysis such as MeDIP-seq or MBD-seq [54] are not appropriate for UC tissue specimens. In contrast, our present results indicate that the contamination of UC-derived carbohydrate polymers in genomic DNA specimens does not significantly affect the bisulfite-based deep sequencing methods of DNA methylome profiling. Methods of preservation of UC and its DNA methylome by refrigeration, freezing, or potentially by drying/lyophilization need to be established in future studies for the conventional use of UC in large-scale population studies.

Genome-wide comparison of DNA methylomes between UC and CB showed clear global differences, which were characterized by the hypo-methylated state of all chromosomes in UC compared to CB (Fig 1A–1C). Differential DNA methylation between UC and CB (both hyper- and hypo-methylated in UC compared to CB) was located mostly in introns or intergenic regions whereas only 10% of DMRs associated promoters of protein-coding genes (Fig 1D). Detailed analysis of promoter DNA methylation revealed that a 5 kbp region upstream of TSS tended to be hypomethylated in UC than CB whereas TSS or its 5 kbp downstream region showed comparable levels of DNA methylation (Fig 1E). In their recent study, Schlosberg *et al*. described the computational tool ME-Class (Methylation-based Gene Expression Classification) for prediction of gene expression based on DNA methylation data and identified the 0.5 kbp—2.5 kbp region immediately downstream of TSS (rather than the region upstream of TSS) as the most informative window of DMRs for prediction of differential gene expression [55]. In our present study, we were unable to integrate the RRBS data with RNA-seq data because of the technical difficulty in isolating intact RNA from UC. Future studies should address whether the DMRs immediately upstream of TSS is liked to differential mRNA expression between UC and CB.

Our gene ontology analysis revealed very strong enrichment of the *HOX* domain-containing transcription factors for both hyper- and hypo-methylated DMRs of UC compared to CB (Figs 2E, 2F, 3E and 3F). Overall, DMRs tended to be enriched with gene ontology terms relevant to mesenchymal tissue development and DNA-binding transcription factors (Figs 2 and 3; S3 Table). Visual inspection of DNA methylation profiles confirmed specific and significant DMRs at promoters of *HOX* genes and several other genes encoding transcription factors involved in body patterning and/or lineage-specific cellular differentiation (Fig 4). These results suggest that the primary differences in DNA methylomes between UC and CB reflect the distinct epigenetic programs regulating development of these fetal tissues through differential expression of transcription factors. Since these DMRs are also associate with developmental anomalies such as clubfoot (*i*.*e*., aberrant bone mineral density; Fig 2A and 2B) or diseases related to cardiovascular, metabolic, or neuronal functions (Fig 3A and 3B), future studies should address whether changes in DNA methylation in UC is associated with these disease conditions better than CB, or *vice versa*.

Sexual dimorphism of autosomal DNA methylation is observed in tissue-dependent manners. Analyzing autosomal DNA methylomes of various tissues of isogenic C57BL/6J mice by RRBS, McCormick *et al*. recently demonstrated that liver shows very strong sexual dimorphism whereas brain and heart showed only modest sex-dependent differential methylation [56]. The gender-dependent DMRs are tissue autonomous, showing very few overlaps between liver, muscle, heart, and spleen. Our present study showed that autosomal DNA methylation of human UC was largely identical between males and females (Fig 5A and 5B). Interestingly, the sex-dependent DMRs in human UC were strongly enriched at protein-coding promoters (Fig 5C and 5D), which was in sharp contrast to that the great majority of mouse sex-dependent DMRs were located in either intergenic or intronic regions [56]. Although the functional significance of the sex-dependent DMRs in human UC is unknown, differences between male and female human UC have been documented. For example, a recent human population-based study involving 856,300 single births recorded in the Medical Birth Registry of Norway has clearly demonstrated that UC of male neonates is significantly longer than female UC and so associated with greater risks of cord knot or entanglement [57]. Since the longer human UC is also significantly associated with gestational diabetes [57], it is interesting to speculate that the sex-dependent DMRs in UC might be involved in the mechanism of UC elongation under gestational diabetes condition, which needs to be determined by future studies.

Folate is an essential water-soluble vitamin for humans and involved in the one-carbon metabolism, which generates S-adenosyl methionine (SAM). As SAM is the common methyl donor substrate in methyl group transfer reactions including DNA cytosine methylation, folate deficiency during early pregnancy increases risks of neural tube defects (*e*.*g*, spina bifida [58]), small fetuses or low birth weight [59–61], and adult-onset disorders [62, 63] often associated with decreased DNA methylation [64]. Our present study did not detect global changes in UC DNA methylation between mothers with high- and low-levels of plasma folate concentration at gestational week 12 (Fig 6A and 6B). However, it is important to note that the plasma folate levels of the low-folate group (7 ± 0.6 ng/mL) was still within the normal range (6–20 ng/mL) although they were significantly lower than the high-folate group (29.7 ± 10.2 ng/mL) (S2 Table). The updated WHO report on vitamin and mineral nutrition information system:VMNIS recommends 3–4 ng/mL plasma folate levels as the cut-off values of folate deficiency (http://apps.who.int/iris/bitstream/handle/10665/162114/WHO_NMH_NHD_EPG_15.01.pdf?sequence=1). Because the low-folate mothers involved in the present study were not in the definitive folate-deficiency state, our data indicate that DNA methylome of human UC was largely unaffected by fluctuations of plasma folate level within the normal range. Surprisingly, our study detected a series of CpG sites whose methylation was far stronger in the low-folate group than the high-folate group, which was counter-intuitive, and these CpGs turned out to be specifically located within two distinct but independent copies of the endogenous retrotransposon LTR12C (Fig 6A–6D). This hypermethylation seemed specific to these two copies of LTR12C because bisulfite pyrosequencing analysis of LTR12C methylation using PCR primers targeting a sequence commonly found in many copies of LTR12C did not detect changes in DNA methylation by the levels or sex (Fig 6E). At present, the reason of the specific hypermethylation of these two copies of LTR12C or its functional significance is unknown. LTR12C is particularly longer and more CpG rich than most other endogenous retrovirus LTRs, and aberrant DNA methylation at or around this class of endogenous retroviruses is often associated with human diseases including cancers [65]. It is interesting to speculate that, in certain types of cells consisting of UC, these two copies of LTR12C might be particularly active in transcription under relatively low-folate conditions, and the mechanisms suppressing active transcription of endogenous retroviruses by heterochromatin formation and subsequent DNA methylation could target them. Further studies will be necessary to examine the potential usefulness of evaluating DNA methylation state at these two copies of LTR12C in the context of toxico-epigenomic studies as well as the molecular mechanisms behind the specific hypermethylation in the low-folate group.

Since the major findings of the present study were based on relatively small numbers of human study subjects without validation using alternative sets of subjects, their applicability to the general public is limited. Future studies are warranted to examine whether our major findings are also observed with other, independent cohorts, involving larger numbers of subjects. The *p*-values of differential methylation at the individual CpG sites in two copies of LTR12C (Fig 6C and 6D) indicate that approximately 18% changes in CpG methylation can be detected with the current sample set (N = 10 for each group of high/low maternal plasma folate) when the SEM is not greater than 5% (Fig 6D). However, if the SEM increases to about 10%, as strong as 32% changes in CpG methylation were needed to obtain statistical significance (Fig 6C). Power analysis agrees with our present observations–namely, when per-group sample size (N) is 10, a 20% change in CpG methylation (from 50% to 70%) will be detected with 0.05 alpha and 80% power under 10% SEM (= 15.8% SD for N = 10). An attempt to detect a smaller change (10%) under the same level of SD (15.8%), alpha, and power, will require 39 samples per group. Detection of small (10%) changes at noisier CpG methylation sites with a doubled SD (31.6%) under the same alpha and power will require 157 samples per group. Thus, the design of our present study was adequate for detection of small (~20%) changes in CpG methylation at sites with relatively small variations (SEM < 10%) or larger (>30%) changes at sites with larger variation (~10% SEM). Future studies should be designed based on the above observations and power analysis for anticipated levels of changes in CpG methylation and available sizes of study cohorts. With the relatively small size of subjects notwithstanding, our present study provides a technical foundation for the use of umbilical cord tissue as surrogate of human newborns. The differences in the DNA methylome at several key developmental genes including the HOX genes are convincing despite the limited number of subjects.

## Conclusions

Our present study has shown that DMRs between human UC and CB are strongly enriched with genes encoding transcription factors involved in developmental body patterning and lineage-specific tissue differentiation. DNA methylomes of male and female UC are largely identical except for DMRs strongly enriched at promoters of protein-coding genes. UC DNA methylome was not strongly affected by fluctuation of plasma folate levels within the normal range during early pregnancy. However, two specific and independent copies of the LTR12C endogenous retrovirus may be particularly sensitive to changes in the folate levels and are hyper-methylated under low-folate conditions. These observations support the usefulness of UC as an alternative epigenetic surrogate of neonates compensating CB for studying environmental and nutritional effects on DNA methylation in human fetuses.

## Supporting information

S1 Table Sample description. (XLSX)

S2 Table Characteristics of the study subjects. (XLSX)

S3 Table Differentially methylated regions detected in promoters from UC-CB analysis. (XLSX)

S1 Fig Quality control data of RRBS. (A) Numbers of uniquely mapped reads of the individual RRBS library. (B) Histograms of CpG coverage of the combined CB or UC libraries for the CB-UC comparison data set. (C) Histograms of CpG coverage of the combined CB or UC libraries for the CB-UC comparison data set.
(TIFF)

S2 Fig Comparison of DNA methylomes of UC and CB between males and females. DNA methylomes of UC and CB obtained from the same newborn (n = 10, 5 males and 5 females) were determined by the reduced representation bisulfite sequencing (RRBS). (A) Principal component analysis of DNA methylomes of UC-males (blue dots), UC-females (green), CB-males (red), and CB-females (orange). The left two cubes show 3-D representations of the same whole-genome analysis data from different viewpoints; the rightmost cube shows data of analysis involving only autosomes. (B-D) X-Y plot profiling of male (x-axis) and female (y-axis) whole-genome (left, red dots) or autosome-only (center, blue dots) DNA methylomes of UCs (panel C), CBs (panel D), or UCs and CBs combined (panel B). The rightmost plots show merged images of the whole-genome and autosome-only profiles; datum points identical between the whole-genome and autosome-only profiles are thus shown in purple. The green ovals highlight locations of female-specific DNA hypermethylation regions detected in the whole-genome analysis but not in the autosomal analysis.
(TIFF)
