# Supplementary figures and images for: DNA methylome of human neonatal umbilical cord: Enrichment of differentially methylated regions compared to umbilical cord blood DNA at transcription factor genes involved in body patterning and effects of maternal folate deficiency or children’s sex
Source: PLoS One. 2019 May 7;14(5):e0214307. doi: 10.1371/journal.pone.0214307 (PMC6504184; doi:10.1371/journal.pone.0214307)

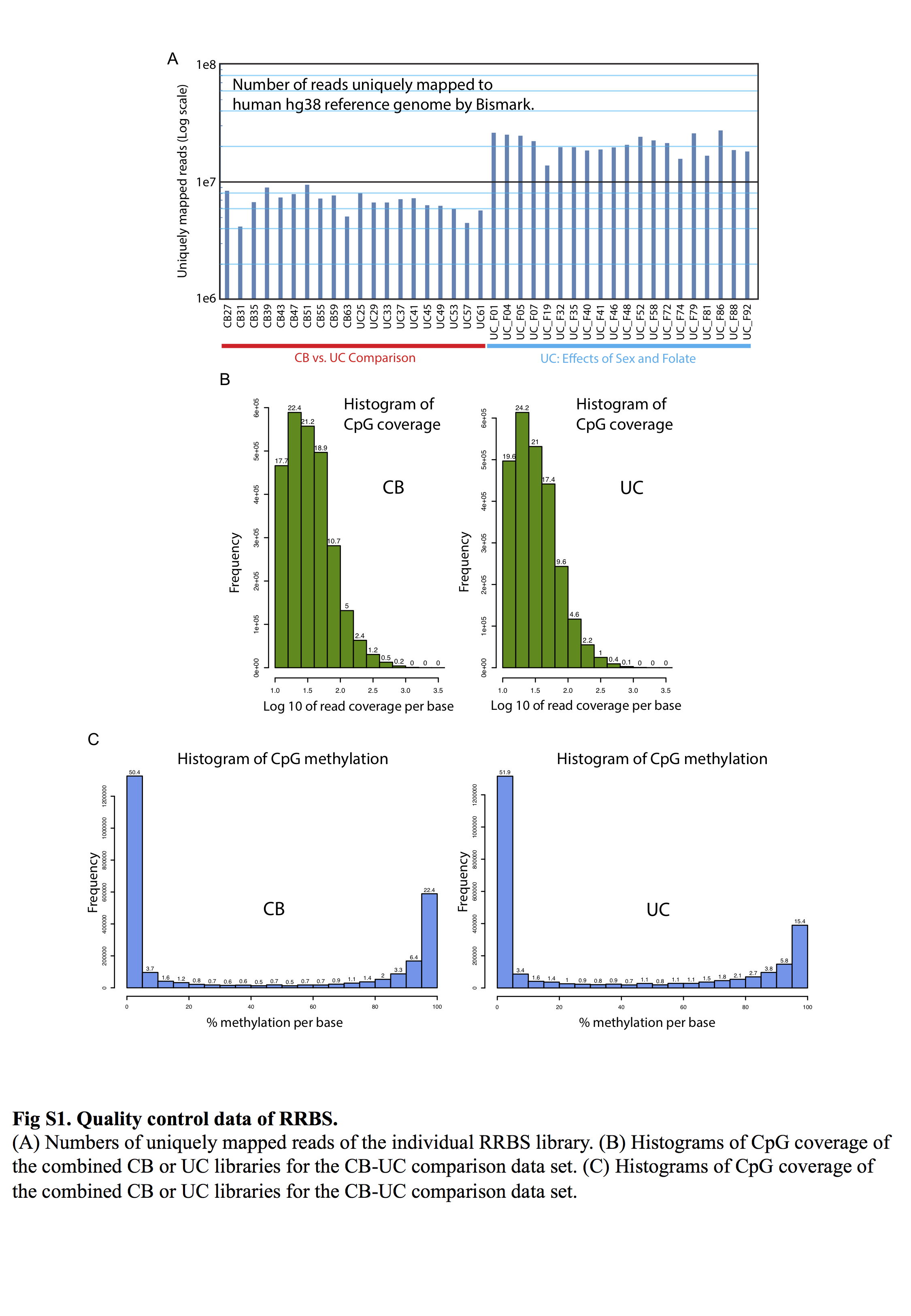

Supplement: S1 Fig — (A) Numbers of uniquely mapped reads of the individual RRBS library. (B) Histograms of CpG coverage of the combined CB or UC libraries for the CB-UC comparison data set. (C) Histograms of CpG coverage of the combined CB or UC libraries for the CB-UC comparison data set. (TIFF) [file pone.0214307.s004.tiff]

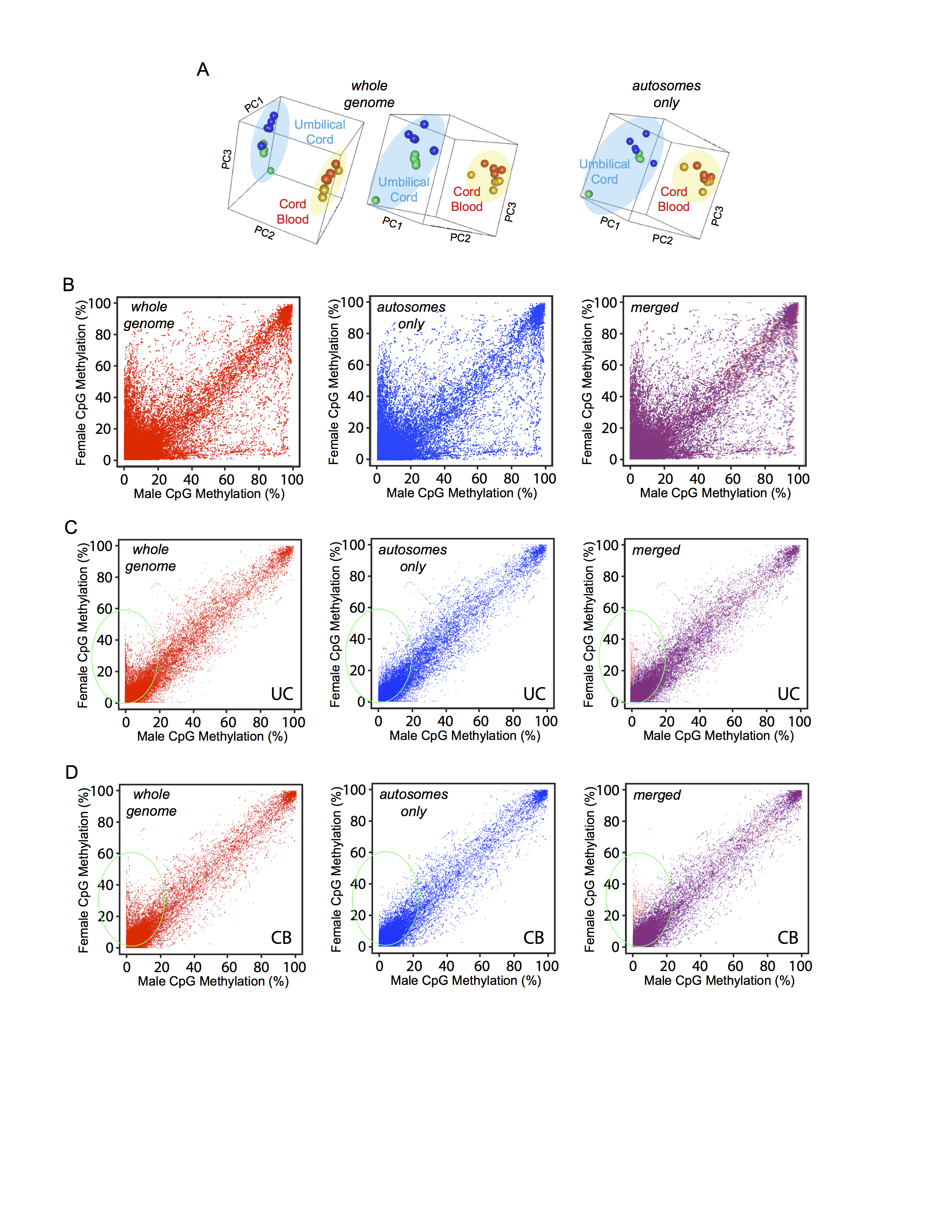

Supplement: S2 Fig — DNA methylomes of UC and CB obtained from the same newborn (n = 10, 5 males and 5 females) were determined by the reduced representation bisulfite sequencing (RRBS). (A) Principal component analysis of DNA methylomes of UC-males (blue dots), UC-females (green), CB-males (red), and CB-females (orange). The left two cubes show 3-D representations of the same whole-genome analysis data from different viewpoints; the rightmost cube shows data of analysis involving only autosomes. (B-D) X-Y plot profiling of male (x-axis) and female (y-axis) whole-genome (left, red dots) or autosome-only (center, blue dots) DNA methylomes of UCs (panel C), CBs (panel D), or UCs and CBs combined (panel B). The rightmost plots show merged images of the whole-genome and autosome-only profiles; datum points identical between the whole-genome and autosome-only profiles are thus shown in purple. The green ovals highlight locations of female-specific DNA hypermethylation regions detected in the whole-genome analysis but not in the autosomal analysis. (TIFF) [file pone.0214307.s005.tiff]
